# Effects of resistance training on serum 25(OH) D concentrations in young men: a randomized controlled trial

**DOI:** 10.1186/s12986-020-00480-w

**Published:** 2020-08-01

**Authors:** Xiaomin Sun, Xiao-Kai Ma, Lin Zhang, Zhen-Bo Cao

**Affiliations:** 1 Global Health Institute, School of Public Health, Xi’an Jiaotong University Health Science Center, Xi’an, 710061 China; 2 School of Kinesiology, Shanghai University of Sport, 399 Chang Hai Road, Shanghai, 200438 China

**Keywords:** Vitamin D, Resistance training, Muscle mass, Physical activity

## Abstract

**Background:**

Previous studies indicated that serum 25-hydroxyvitamin D [25(OH)D] concentrations are positively associated with physical activity levels independent of sun exposure. However, the effect of resistance training on serum 25(OH) D concentrations remains unclear. Thus, this study aimed to examine the effect of chronic resistance training on serum 25(OH) D concentrations and determine whether 25(OH) D concentration variations are influenced by body composition changes.

**Methods:**

Eighteen young men aged 19–39 years were randomly divided into a 12-week resistance training group (RT, *n* = 9) and non-exercise control group (CON, *n* = 9). The trial was undertaken in Shanghai University of Sport in Shanghai, China. Randomization and allocation to trial group were carried out by a central computer system. Serum 25(OH) D and intact parathyroid hormone concentrations were measured using commercially available enzyme-linked immunosorbent assay kits. Body composition was measured by dual-energy X-ray absorptiometry.

**Results:**

The average serum 25(OH) D concentrations were 26.6 nmol/L at baseline. After the 12-week intervention program, serum 25(OH) D concentrations significantly increased in both groups. Serum 25(OH) D concentrations at midpoint (6-week) increased significantly only in the CON group (*P* < 0.01). From training midpoint to endpoint, a significantly greater increase in serum 25(OH) D concentrations was noted in the RT group (P-interaction = 0.043); 25(OH) D concentration changes (end-pre) were negatively related to fat-free mass (mid-pre) (*r* = − 0.565, *P* = 0.015) and muscle mass (mid-pre) (*r* = − 0.554, *P* = 0.017).

**Conclusions:**

There were no beneficial effects of the 12-week resistance training on serum 25(OH) D concentration in vitamin D deficient young men, and an indication that seasonal increase in serum 25(OH) D concentrations during the early phase of resistance training was transiently inhibited, which may partly be attributed to resistance training-induced muscle mass gain.

**Trial registration:**

Chinese Clinical Trial Registry, ChiCTR2000030876. Registered 16 March 2020 - Retrospectively registered, http://www.chictr.org.cn/showproj.aspx?proj=50504.

## Introduction

Circulating 25-hydroxyvitamin D [25(OH)D] is the major form of vitamin D, which is used in vitamin D status assessment. Accumulated studies indicated that vitamin D not only regulates calcium and phosphorus metabolism but also plays an important role in modifying cardiovascular diseases risk, and improving immune function and physical fitness [1–4].

Previous studies indicated that higher levels of physical activity or cardiorespiratory fitness were related to higher circulating 25(OH) D concentrations [5–7]. Although individuals who were physically active outdoors are more likely to have higher circulating 25(OH) D due to increased sun exposure [8, 9], this relationship was still observed after adjustment for sunlight exposure [6, 10]. Scragg et al. [5] found that higher levels of physical activity were associated with higher serum 25(OH) D concentrations not only during summer but also during winter, when the vitamin D synthesis from sun exposure is extremely limited [11]. Consistently, several intervention studies suggested that endurance exercise could increase circulating 25(OH) D or prevent its seasonal reduction [12–14]. These findings indicated that physical activity could directly increase 25(OH) D concentrations.

Resistance training (RT) promotes health benefits through increased skeletal muscle mass and qualitative adaptations [15]. In addition to adiposity tissue, muscle tissue was suggested to serve as an another important site to store 25(OH) D, and then it returns to the circulating when needed [16, 17]. Nevertheless, Makanae and colleagues reported that intramuscular expression of CYP27B1, which catalyses the hydroxylation and activation of 25(OH) D, was significantly increased at 1 h and 3 h after resistance exercise, which may enhance local vitamin D metabolism in skeletal muscle [18]. It is well known that compared with endurance exercise, RT could not only reduce fat mass, but also it is more effective in increasing muscle mass and strength [19, 20], which may largely affect vitamin D metabolism. However, whether serum 25(OH) D concentrations are affected by RT remains unclear.

This study aimed to evaluate the effect of chronic resistance training on serum 25(OH) D concentrations and to determine whether the effects are attributable to changes in body composition in healthy young men.

## Methods

### Participants

Eighteen healthy men with no chronic diseases (aged 19 to 39 years) were included in this study, which was conducted from March to July 2016. Participants using vitamin D supplements or sunscreen regularly were excluded. Assessments were performed at 0 week (pre), 6 weeks (midpoint), and 12 weeks (endpoint). All subjects provided informed consent for inclusion before they participated in the study. The study was conducted in accordance with the Declaration of Helsinki, and the protocol was approved by the Ethics Committee of Shanghai University of Sport (2016001). The trial has been retrospectively registered at Chinese Clinical Trial Registry website (ChiCTR2000030876).

### Experimental protocol

Eighteen participants were randomly allocated to the following two groups by using a computer-generated random number sequence: resistance training group (RT) and no-exercise control group (CON). Participants in the RT group were instructed to exercise 2–3 times a week in a gymnasium. A supervised training session was performed in the afternoon between 1630 h and 2000 h during the 12 weeks of progressive resistance training. In the first 2 weeks, the participants were taught how to properly perform each movement. The resistance workload gradually changed from light to heavy, and the participants were instructed to complete 10 repetitions for each set of the following exercises: leg press, preacher curl, chest press, leg curl, and iso row, with 60–120-s rest period between two sets; additionally, they also performed three sets of 15–25 repetitions of abdominal crunch and back extension with their own body weight, with a 60–120-s rest period between two sets. At the first training session of the third week, the participants were instructed to perform strength testing, and 12 repetitions maximum (12RM) for each exercise were performed, except back extension and abdominal crunch. The workload for the next resistance training was calculated from the participants’ previously determined 12RM. The participants were encouraged to perform back extension and abdominal crunch exercises 30 times from the first training session of the third week. When the strength training equipment at our gymnasium had been upgraded at the eighth week of training, the resistance training program was revised to include the following: leg extension, standing one-arm cable curl, standing cable chest press, leverage shoulder press, leverage high row, back extension, and abdominal crunch. These new components of the resistance training program were selected based on the principle of using similar muscle groups. The order of resistance exercise was randomized; however, training of the same muscle groups continuously was avoided. The participants were instructed not to undertake any formal exercise or change their levels of general physical activity and dietary habits.

### Anthropometric measurements

Height was measured with the participants wearing light clothing and being barefoot (HK6000-ST, HENGKANGJIAYE Technology Co., Ltd. CHN). Dual-energy X-ray absorptiometry (DXA Prodigy, GE Lunar Corp., Madison, WI, USA) was used for the measurement of whole and regional body composition, including total body mass, muscle mass, fat mass, fat percentage, and bone mineral density. Body mass index was calculated as body mass (kg) divided by height (m^2^). Fat-free mass (FFM) was calculated as body mass minus fat mass.

### Physical activity and sun exposure

Physical activity was measured using Actigraph GT3X+ accelerometers (ActiGraph, LLC, Pensacola, FL, USA). Participants were instructed to wear the accelerometers on their right hip at all times, except during water activities (swimming, showering) or sleeping, for 7 consecutive days at baseline. A wear time of ≥480 min/day was used as the criterion for a valid day, and ≥ 2 weekdays and 1 weekend day was used as the criteria for a valid 7-day period of accumulated data. Non-wear time was defined as consecutive periods of ≥60 min of zero accelerometer counts and excluded from the analyses. Data were recorded in 60-s epochs. A pragmatic cutoff of ≥2690 cpm was used to categorize moderate-vigorous physical activity time [21, 22]. The 7-day data collection for all subjects was fixed within 2 weeks. Outdoor time from 0900 h to 1600 h for 7 consecutive days was recorded and used as an alternative for sun exposure in the analysis.

### Blood analysis

Blood samples were obtained after at least an 8-h overnight fasting period and subsequently centrifuged at 3000 rpm at 4 °C for 10 min. Serum was collected and stored at − 80 °C until analysis. Serum 25(OH) D and intact parathyroid hormone (iPTH) concentrations were measured in duplicate using commercially available enzyme-linked immunosorbent assay kits (25(OH) D, IDS, Bolton, UK; iPTH, DRG, Marburg, Germany) according to the manufacturer’s instructions. The intra-assay and inter-assay coefficients of variation were 9.1 and 7.7%, respectively, for serum 25(OH) D and 9.6 and 3.6%, respectively, for iPTH. Vitamin D deficiency is defined as 25(OH) D concentration < 50 nmol/L, and vitamin D insufficiency as 25(OH) D concentration < 75 nmol/L [23].

### Statistical analysis

All statistical analyses were performed using IBM SPSS 24.0 for Windows (SPSS Inc., Chicago, IL, USA). Data were assessed for normality using a Kolmogorov-Smirnov test prior to all statistical analyses. Normally distributed variables were presented as mean ± standard deviation, and non-normally distributed variables were presented as median (interquartile range), unless otherwise indicated. The Student t test (for normally distributed variables) or the Mann-Whitney *U* test (for non-normally distributed variables) was used to evaluate the differences between groups. Two-factor repeat-measured analysis of variance (ANOVA) (group × time) was used to determine the effect of exercise on 25(OH) D, iPTH, and body composition indicators. A post hoc test with Bonferroni correction was performed to identify statistically significant differences among the mean values when a considerable interaction was identified. Pearson correlation and partial correlation coefficients were computed between 25(OH) D concentration changes and body composition indicators. The level of statistical significance was set at *P* < 0.05.

## Results

The participants’ characteristics and blood parameters at baseline are presented in Table 1. The average serum 25(OH) D concentrations were 26.6 nmol/L, and all participants showed vitamin D deficiency. No significant difference in either body composition indicators or blood parameters was found between the groups (*P* > 0.05).

**Table 1:** Participant characteristics according to groups at baseline

Variable	Resistance training group (RT) (***n*** = 9)	Non-exercise control group (CON) (***n*** = 9)
Age (y)	24.2 ± 3.1	26.7 ± 6.2
Height (cm)	1.74 ± 0.03	1.75 ± 0.06
Weight (kg)	68.5 ± 9.7	68.7 ± 7.6
BMI (kg/m^2^)	22.5 ± 2.9	22.3 ± 1.9
FFM (kg)	54.2 (49.2 –56.4 )	57.5 (51.1 –58.8 )
Muscle mass (kg)	51.4 (46.6 –53.3 )	54.9 (48.4 –55.8 )
Body fat percentage (%)	21.9 ± 6.9	18.8 ± 6.0
Arms fat percentage (%)	18.7 ± 7.1	14.4 ± 5.1
Legs fat percentage (%)	20.5 ± 5.6	18.1 ± 5.2
Trunk fat percentage (%)	25.1 ± 8.3	21.6 ± 7.3
BMD (g/cm^2^)	1.16 ± 0.07	1.18 ± 0.07
25(OH) D (nmol/L)	26.9 ± 4.7	26.2 ± 4.1
iPTH (pg/mL)	54.9 ± 19.8	44.6 ± 23.8
MVPA (min/day)	69.8 ± 32.2	65.8 ± 16.3
Sun exposure (min/day)	40.0 ± 27.9	50.0 ± 30.7

Values are presented as mean ± standard deviation, median (interquartile range), or percentage

Student *t* test (for normally distributed variables) or the Mann-Witney *U* test (for nonnormally distributed variables) was used to evaluate the differences. Significant difference, *P* < 0.05

*BMI* body mass index, *FFM* fat free mass, *25(OH) D* 25-hydroxyvitamin D, *iPTH* intact parathyroid hormone, *MVPA* moderate-vigorous physical activity

As shown in Table 2, significant interactions of group × time were found for FFM (*P* = 0.004) and muscle mass (*P* = 0.003). After resistance training, the FFM values (mid-pre, 2.7%, *P* = 0.003; end-pre, 3.0%, P = 0.004) and muscle mass (mid-pre, 3.0%, *P* = 0.002; end-pre, 3.4%, *P* = 0.004) significantly increased at the midpoint and endpoint compared to the baseline values, whereas no significant changes were observed in the CON group. In addition, we observed borderline significant interactions in body fat percentage, arm fat percentage, leg fat percentage, and trunk fat percentage (Table 2).

**Table 2:** Characteristics of the participants at baseline, midpoint and endpoint

Variable	Resistance training (RT) group (*n* = 9)			Non-exercise control (CON) group (*n* = 9)			*P*		
	Baseline	Midpoint	Endpoint	Baseline	Midpoint	Endpoint	Time	Group	Time × Group interaction
Weight (kg)^*a*^	68.4 ± 3.1	68.6 ± 3.1	68.5 ± 3.0	68.8 ± 3.1	68.7 ± 3.1	68.6 ± 3.0	0.156	0.958	0.762
BMI (kg/m^2^)^*a*^	22.6 ± 0.9	22.6 ± 0.9	22.6 ± 0.8	22.3 ± 0.9	22.2 ± 0.9	22.2 ± 0.8	0.273	0.768	0.455
FFM (kg)^*a*^	52.5 ± 1.7	53.9 ± 1.8^c^	54.1 ± 1.8^c^	55.9 ± 1.7	56.1 ± 1.8	55.8 ± 1.8	0.347	0.341	**0.004**
Muscle mass (kg)^*a*^	49.7 ± 1.6	51.2 ± 1.7^c^	51.4 ± 1.7^c^	53.1 ± 1.6	53.2 ± 1.7	53.0 ± 1.7	0.399	0.348	**0.003**
Body fat percentage^*a*^	22.4 ± 2.0	20.6 ± 1.9	20.1 ± 2.0	18.3 ± 2.0	18.0 ± 1.9	18.3 ± 2.0	0.643	0.327	0.058
Arms fat percentage^*a*^	19.3 ± 1.8	17.9 ± 1.8	17.2 ± 1.7	13.7 ± 1.8	13.4 ± 1.8	13.6 ± 1.7	0.687	0.091	0.077
Legs fat percentage^*a*^	20.8 ± 1.7	19.3 ± 1.6	19.2 ± 1.7	17.7 ± 1.7	17.2 ± 1.6	17.6 ± 1.7	0.846	0.378	0.064
Trunk fat percentage^*a*^	25.7 ± 2.5	23.6 ± 2.4	22.8 ± 2.4	21.1 ± 2.5	20.8 ± 2.4	21.1 ± 2.4	0.484	0.397	0.075
BMD (g/cm^2^)^*a*^	1.16 ± 0.02	1.16 ± 0.02	1.17 ± 0.03	1.18 ± 0.02	1.18 ± 0.02	1.20 ± 0.03	**0.016**	0.538	0.235
25(OH) D (nmol/L)^*b*^	26.0 ± 1.8	29.2 ± 2.7	36.7 ± 2.7^cd^	27.2 ± 1.8	37.2 ± 2.7^c^	40.7 ± 2.7^cd^	0.807	0.261	**0.025**
iPTH (pg/mL)^*b*^	61.9 ± 8.3	58.7 ± 6.1	63.4 ± 5.6	37.6 ± 8.3	48.3 ± 6.1	51.5 ± 5.6	0.110	0.131	0.242
Sun exposure (min/day)	40.0± 9.8	76.5 ± 13.5	63.8 ± 15.2	50.0 ± 9.8	73.5 ± 13.5	50.5 ± 15.2	**0.015**	0.889	0.493

Data are presented as the adjusted mean ± SE. *BMI* body mass index, *FFM* fat free mass, *BMD* bone mineral density, *25(OH) D* 25-hydroxyvitamin D, *iPTH* intact parathyroid hormone. ^*a*^ Adjusted by age, MVPA. ^*b*^ Adjusted by age, MVPA, baseline test time, changes of sun exposure and body fat percent from pre to end. ^c^Change is significantly different from the baseline value within a subgroup. ^d^ Change is significantly different from the endpoint to midpoint within a subgroup. Boldface indicates significance (*P* < 0.05)

**Fig. 1:**
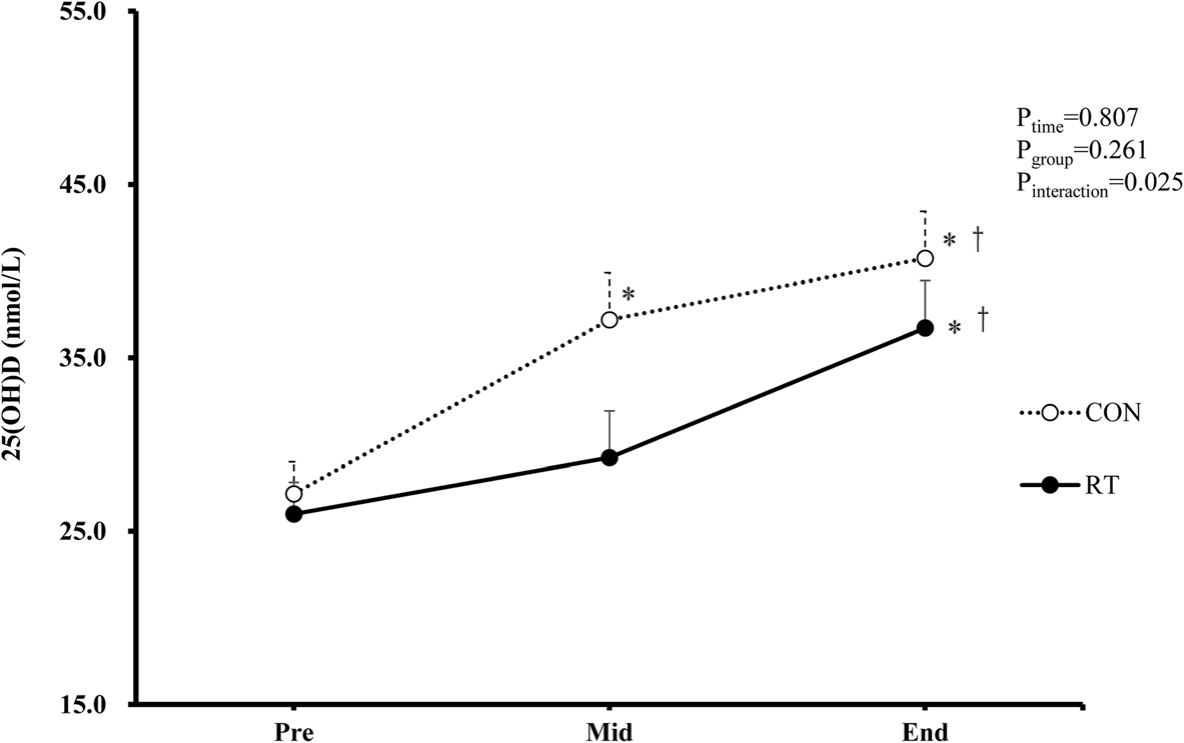
Effects of resistance training on serum 25(OH) D concentrations. The analysis was adjusted according to age, MVPA, and changes in sun exposure and body fat percentage (End-Pre). 25(OH) D, 25-hydroxyvitamin D; RT, resistance training; CON, non-exercise control; MVPA, moderate to vigorous physical activity. *, compared with pre, *P* < 0.05; †, compared with mid, *P* < 0.05

As shown in Table 2 and Fig. 1, a significant group × time interaction was observed for serum 25(OH) D concentrations (*P* = 0.025). After the 12-week intervention, the mean serum 25(OH) D concentrations significantly increased to 36.7 ± 2.7 nmol/L in the RT group and 40.7 ± 2.7 nmol/L in the CON group. Moreover, in the CON group, higher serum 25(OH) D concentrations were observed at midpoint compared with the values at baseline (*P* < 0.01), whereas no notable differences were found in the RT group. From midpoint to endpoint, although serum 25(OH) D concentrations significantly increased in both groups, the increase was significantly greater in the RT group (25.8%) than in the CON group (9.4%) (P-interaction =0.043).

**Fig. 2:**
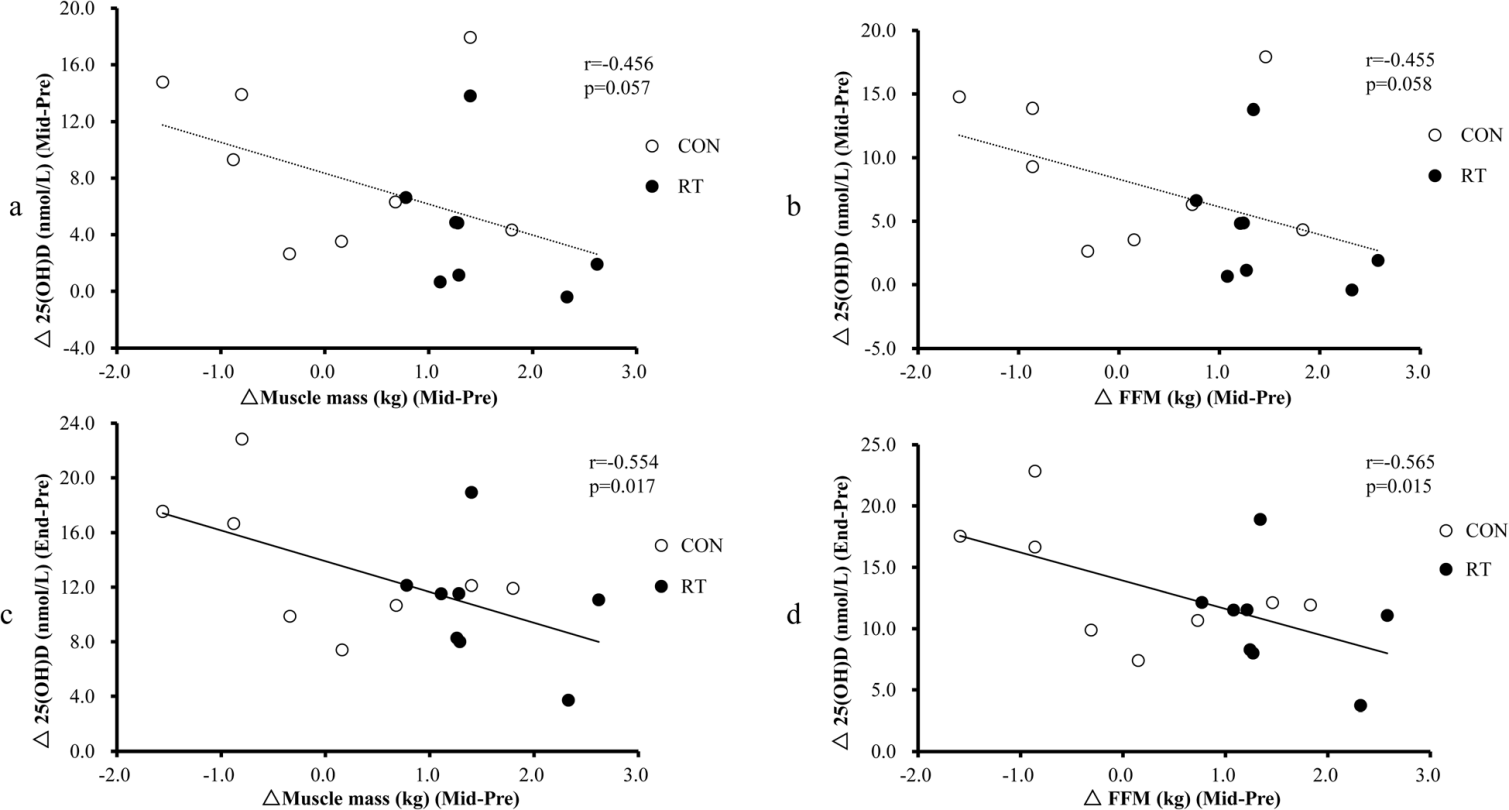
Correlation of changes in 25(OH) D concentrations with changes in muscle mass and FFM. 25(OH) D, 25-hydroxyvitamin D; FFM, fat-free mass; RT, resistance training; CON, non-exercise control

Correlation analyses were performed to identify the factors associated with the changes in 25(OH) D concentrations (Table 3). 25(OH) D concentration changes (end-pre) were negatively related to FFM (mid-pre) (*P* = 0.015) and muscle mass (mid-pre) (*P* = 0.017) changes (Fig. 2); a borderline association was observed between 25(OH) D concentration changes (mid-pre) and FFM (mid-pre) (*P* = 0.058) and muscle mass (mid-pre) (*P* = 0.057) changes. In addition, significant correlations of 25(OH) D concentration changes (end-pre) with FFM (mid-pre) (*P* = 0.039) and muscle mass (mid-pre) (*P* = 0.043) changes were observed after adjustment for group, age, and changes in sun exposure (end-pre), body weight (end-pre), and body fat percentage (end-pre).

**Table 3:** Correlations between changes in indicators of body composition and changes in serum 25(OH) D concentrations

Variables	25(OH)D_mid-pre_		25(OH)D_end-pre_	
	r	*P*	r	*P*
Body fat percentage				
mid-pre	0.014	0.956	0.101	0.690
end-mid	−0.217	0.387	−0.004	0.986
end-pre	−0.090	0.722	0.064	0.800
FFM				
mid-pre	−0.455	0.058	**−0.565**	**0.015**
end-mid	0.121	0.632	0.037	0.883
end-pre	−0.289	0.244	−0.424	0.080
Muscle mass				
mid-pre	−0.456	0.057	**−0.554**	**0.017**
end-mid	0.142	0.575	0.037	0.884
end-pre	−0.280	0.261	−0.415	0.087

*25(OH) D* 25-hydroxyvitamin D, *FFM* fat-free mass

## Discussion

In this study, we found that the seasonal increase in serum 25(OH) D concentrations in young men was transiently inhibited during the early phase of RT; subsequently, serum 25(OH) D concentrations increased in both RT and CON groups, and the increase was observed to be greater in RT group than in CON group. The transient inhibition could be partly attributed to muscle mass and fat free mass gain induced by RT in the early phase.

Previous studies have reported significant positive associations of serum 25(OH) D concentrations with physical activity levels independent of sun exposure [6, 10]. Bell et al. [24] found that 25(OH) D concentrations were higher in participants who had engaged in regular muscle-building exercise for at least 1 year compared to those in the control group. Consistently, our recent studies also observed that exercise training could increase serum 25(OH) D concentrations or prevent its seasonal reduction in young and old men [13, 14]**.** However, the effect of RT on serum 25(OH) D concentrations remains unclear.

Compared with endurance training, RT is more effective in increasing muscle mass and strength [24], which was also supported by our results. In the present study, we observed that after resistance training, body mass was reduced, while FFM and muscle mass were significantly increased at the midpoint and endpoint compared to the baseline values, whereas no significant changes were observed in CON group.

In addition to adiposity tissues, muscle tissues are also served as another important storage for 25(OH) D, which could enable optimal vitamin D status to maintain its physiological function during winter months [17]. Makanae and colleagues observed that intramuscular CYP27B1 was significantly increased after resistance exercise in skeletal muscle, which may enhance local vitamin D metabolism [18]. Srikuea et al. [25] also found that the CYP27B1 expression in the muscle increased significantly during skeletal muscle regeneration from injury. Thus, we speculated that the transient inhibition in serum 25(OH) D levels in RT group during the early phase of resistance training could be partly due to absorption of serum 25(OH) D in the increasing muscle mass and its use in muscle activity. 25(OH) D consumption in muscle may induce a compensatory increase in serum 25(OH) D concentrations; however, the concentrations were still lower than that in control group, which may due to the lack of vitamin D storage and short-term intervention period. Previous studies report that serum 25(OH) D concentrations gradually increase from around March to August [26]. This could partly explain the reason why serum 25(OH) D concentration was transiently inhibited in early Spring and followed by a marked increase in early Summer in our study. Future studies that aim at exploring the mechanism underlying the effect of resistance training on serum 25(OH) D concentrations are warranted.

This study has several limitations. First, our study included only men with relatively lower serum 25(OH) D concentrations. Therefore, it remains to be established whether these findings are applicable to other subjects with higher 25(OH) D concentrations. Second, considering the seasonal variation of serum 25(OH) D concentration, the findings in our study have seasonal limitations. Third, at baseline resistance training group had relatively lower values of muscle mass and body fat percentage, although they are not statistically significant, which should be interpreted with caution. Fourth, we couldn’t observe any relationship between muscle mass and 25(OH) D at three points, which may due to the small sample size. However, the muscle mass changes were significantly related with 25(OH) D concentration changes. Finally, although our sample size was relatively small, a post hoc power calculation would still give us a 90% chance, with an effect size of 0.58, to demonstrate the interaction effect of serum 25(OH) D concentrations, which was considered statistically significant at *P* < 0.05 at the endpoints.

## Conclusions

In summary, serum 25(OH) D concentrations in vitamin D deficient young men was observed to be transiently inhibited during the early phase of resistance training and subsequently increased in the later phase. This finding could be partly attributed to the muscle mass and fat free mass gain induced by resistance training during the early phase. Our findings indicated that adequate administration of oral supplements or increased daily dietary vitamin D intake are encouraged to increase serum 25(OH) D concentrations, especially for those who exercise regularly.

## Data Availability

The datasets used and/or analysed during the current study are available from the corresponding author on reasonable request.

## Abbreviations

25(OH)D: 25-hydroxyvitamin D
BMD: Bone mineral density
BMI: Body mass index
FFM: Fat-free mass
iPTH: parathyroid hormone
MVPA: Moderate and vigorous physical activity
RM: Repetition maximum
